# Is the association between graded sickness absence and return to work confounded by health? A longitudinal cohort study from the Norwegian neck and back registry

**DOI:** 10.1186/s12889-025-22368-1

**Published:** 2025-03-30

**Authors:** Ingvild Bardal, Nils Abel Prestegård Aars, Samineh Sanatkar, Sharon AM Stevelink, Oda Lekve Brandseth, Beate Brinchmann, Arnstein Mykletun

**Affiliations:** 1 Centre for Work and Mental Health, Nordland Hospital Trust, Bodø, Norway; 2 Department of Community Medicine, UiT- The Arctic University of Norway, Tromsø, Norway; 3 Black Dog Institute, School of Psychiatry, UNSW Sydney, Randwick, Australia; 4 Department of Psychological Medicine, Institute of Psychiatry, Psychology and Neuroscience, King’s College London, London, UK; 5 King’s Centre for Military Health Research, Department of Psychological Medicine, Institute of Psychiatry, Psychology and Neuroscience, King’s College London, London, UK; 6 Centre for Population Health, Research Department, Division for Mental Health, Haukeland University Hospital, Bergen, Norway; 7 Division for Health Services, Norwegian Institute of Public Health, Oslo, Norway

**Keywords:** Musculoskeletal pain, Sick leave, Return to work, Graded sickness absence, Fit note

## Abstract

**Background:**

Musculoskeletal disorders (MSD) are among the leading causes of sickness absence (SA) and disability. Graded sickness absence (GSA) as an alternative to full time sickness absence (FSA) has been implemented in the Nordic countries to promote return to work (RTW) and prevent disability, similar to the Fit Note in the UK. However, the evidence of the effects of GSA on RTW is limited. FSA is plausibly associated with more health problems than GSA. The aim is to investigate if the hypothesized benefits of GSA over FSA on RTW is confounded by health in a cohort of sick listed patients referred to secondary care due to MSD.

**Methods:**

Data was obtained from the Norwegian Neck and Back Register and the Norwegian Labour and Welfare Administration. Poisson regression was used to estimate the association of GSA versus FSA on RTW at 12 months after assessment, with and without adjustment for measures of symptom severity.

**Results:**

A total of 3371 patients were on GSA (*n* = 1671, 49.6%) or FSA (*n* = 1700, 50.4%) at baseline. Patients on FSA reported more severe symptoms than those on GSA on all measures, and detailed analysis of GSA indicated more severe symptoms with higher SA levels. Patients on GSA had higher rates of RTW at 12 months follow up than patients on FSA (unadjusted RR = 1.29, 95% CI 1.22—1.37), and the association remained in the fully adjusted model (RR = 1.19, 95% CI 1.12–1.26). We found an association between levels of GSA and RTW rates, with more work being associated with higher RR for RTW.

**Conclusions:**

Among sick listed patients referred to secondary care due to MSD, GSA is associated with higher rates of RTW than FSA. Some of the beneficial association between GSA and RTW is confounded by higher symptom levels in FSA than GSA patients, but most of the benefit remains after adjusting for symptom severity. Mechanisms for the benefit of GSA remains unknown.

**Supplementary Information:**

The online version contains supplementary material available at 10.1186/s12889-025-22368-1.

## Background

Musculoskeletal disorders (MSD) are among the most common diagnoses for sickness absence (SA) and disability. Globally, MSD make up about two thirds of the estimated rehabilitation needs of working adults and are the main contributors to years lived with disability [1–3]. The social security of receiving SA benefits when ill is a positive trait of welfare states, but it also presents challenges for both society and als. It is well established that prolonged SA can have a negative impact on a person’s health, and it is a risk factor for a permanent exit from the labour market [4]. People who do not participate in the workforce appear to have more physical and mental health problems than those who do [5]. It has also been shown that health can improve when people re-enter the workforce [6]. A crucial challenge is therefore to disrupt the process of prolonged SA before it progresses into permanent disability, and graded sickness absence (GSA) has been proposed as a way to limit the negative side effects of prolonged SA (7–8).

All industrialised countries share a common interest in reducing SA and promoting a healthy and productive workforce. One strategy is to instigate a framework where SA and disability is not a binary option, but rather related to the individual’s grade of work ability. Variations of this practice can be found in several OECD countries, though concepts and terminology vary [2]. In the Nordic countries GSA, also called partial sick leave, has been implemented as a legal option to prevent high SA rates and labour market exclusion [9]. The Fit Note, which has similar intention, was implemented in England, Wales and Scotland in 2010 [10]. However, the Fit Notes have been relatively sparely used and there are studies suggesting the arrangement has been incompletely researched and not implemented as intended [11, 12]. In Norway, GSA has been part of policy since 2011 and represented about one-fifth of all SA cases in 2020. The SA rate in Norway is currently about 6,5%, with one in three cases certified due to MSD [13]. According to the Norwegian National Insurance Act, GSA should be preferred to full time sickness absence (FSA) if feasible, when an individual assessment by a medical doctor indicates that SA is warranted [14]. GSA should be prescribed if the patient can perform their regular or new tasks to some extent, with adjustments made through workplace accommodations if necessary [14]. This involves an assessment of the patient’s work capacity in relation to their workplace, as well as the potential for accommodations. The doctor prescribing SA must specify either FSA or GSA, and GSA will be decided as a percentage of planned working hours. The lowest level at which GSA can be certified is 20% [14]. However, the employee on SA has the option to work more than the specified SA percentage. In the clinical guidelines for MSD, it is emphasised that resuming activity, including work, can be beneficial for the prognosis of the disease [15]. In this context, prescribed SA could be viewed as an effective form of treatment of diseases and disabilities [16].

Although GSA is implemented in many countries due to presumed beneficial effects on return to work (RTW), the evidence is limited and somewhat inconsistent. A systematic mapping review on GSA stressed that there are no randomized controlled trials and questioned the observational data and ambivalent findings, concluding that further evidence is needed [9]. It has, for example, been reported that GSA may delay RTW compared to FSA in MSD patients [17], and a systematic review suggests that working while ill is a risk factor for future SA and decreased self-rated health [18]. It has been shown that multiple factors beyond medical conditions can contribute to sick leave [19, 20]. Work- related factors, including physical, organizational, psychological and societal aspects of work can affect health [21], and the possibilities for participating in graded work despite MSD will, in addition to the patient’s work capacity, depend on the adaptability of the workplace. The RTW process following long term SA should be seen as a complex process influenced by several factors, both at the patient level and at the system level, including labour market, work environment factors, and SA compensations systems. For instance, Brendbekken and colleagues found that only duration of SA at baseline was associated with RTW at 12 months follow up in patients with chronic musculoskeletal pain [22]. Within the field of occupational health, comparisons across studies are challenged not only by differences in welfare systems and benefit schemes between countries, but also by the varying definitions of the outcome of work across studies. For example, in a systematic mapping review of effects of GSA versus FSA on SA and work participation, the authors identified 15 different outcome measures of RTW (e.g. work participation, SA duration, disability, social welfare benefits) in the 13 studies they included [9]. A shared limitation of existing studies on GSA and RTW is however the lack of adjustment for self-reported health. This is an obvious candidate for confounding in the alleged benefits of GSA over FSA on RTW [9], wherein a higher rate of RTW among patients on GSA is simply attributed to better health in this group. Although some studies [17, 23, 24] include adjustments for diagnoses, this does not reflect severity of conditions and symptoms, increasing the risk of residual confounding.

In order to address this knowledge gap, we aim to examine the role of self-reported health as a potential confounder in the association between GSA and RTW among patients on SA referred to multidisciplinary physical medicine outpatient clinics due to MSD.

## Methods

### Study design, setting and participants

We used observational longitudinal registry data from a cohort of adult patients attending multidisciplinary physical medicine outpatient clinics in Norway between 2016 and 2022. The patients were referred by general practitioners with an indication for specialist health care.

Patient survey data was obtained from the Norwegian Neck and Back Registry (NNRR), a national medical quality register which routinely collects health data from patients who are treated at the multidisciplinary physical medicine outpatient clinics in Norwegian hospitals. Prior to consultation at the clinic, patients complete a questionnaire covering sociodemographic, lifestyle and health measures, while clinicians record diagnosis after the consultation [25]. Information on SA benefit status for the analyses was retrieved from the Norwegian Labour and Welfare Administration (NAV), a national administration registry for benefit records, using the social security number for linkage.

**Fig. 1:**
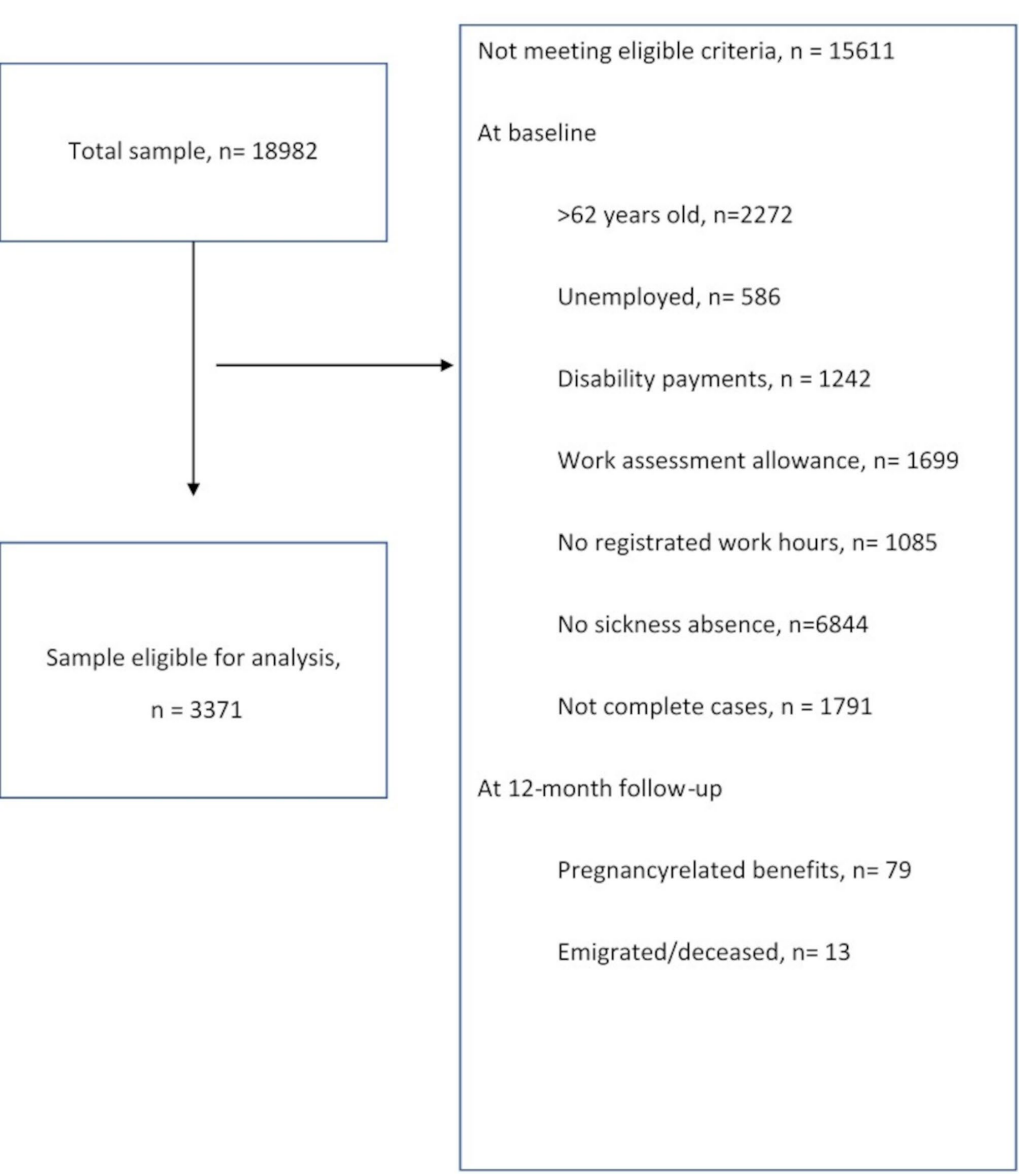
Flow diagram illustrating the selection process

A total of 17,895 patients were assessed for eligibility. Patients aged more than 62 years, emigrated/deceased, currently unemployed, on Work Assessment Allowance (WAA), receiving Disability Payments (DP), on pregnancy- related benefits or with no working hours registered at baseline where excluded. The exclusion criteria were chosen based on their strong association with the outcome of RTW within the observation period. Finally, we also excluded patients with missing data on key variables and patients not currently on SA. This resulted in a final baseline sample of 3371. A flow diagram illustrating the selection process is presented in Fig. 1.

All residents of Norway are covered by the public insurance system, which provides healthcare benefits through the National Insurance Scheme, administered by NAV. When a worker is sick listed by a doctor, NAV`s workers ‘compensation program covers 100% of lost income up to 52 weeks, with employers covering the first 16 days. After one year, irrespective of GSA or FSA in the period, NAV provides long-term rehabilitation benefits (WAA) or DP, covering about 66% of the worker`s previous income. These long-term benefits can be combined with work but is only available if the disability reduces work ability by at least 50% [14].

### Variables and data measurement

#### Exposure

SA status at the time of assessment at the physical medicine outpatient clinic was retrieved from NAV registers. Participants were categorised as either GSA total (20–95% SA) or FSA (100% SA). Further, the patients were categorised into three different levels of GSA: GSA with low SA (SA grading < = 40%), GSA with middle SA (SA grading < 40% and < = 60%), and GSA with high SA (SA grading > 60% and < 100%).

#### Outcome

The outcome of RTW at 12 months after baseline was determined in NAV register data, thus avoiding attrition as the registry was complete with no missing information for any patient. RTW was defined as not being a recipient of SA benefit (neither GSA or FSA) or other long-term health benefits such as DP and WAA at the day of 12 months follow-up.

#### Adjustment variables

All adjustment variables were obtained from NNRR data at baseline, except duration of SA at baseline which was obtained from NAV data.

#### Sociodemographic variables

Age was included as a categorical variable in Table 1 and as a continuous variable in the analyses. Sex was reported as male or female. Marital status was consolidated into a binary variable. Education level was reported in five levels, from primary school to university over four years. Occupation was reported by patients in open questions, which was then classified using the International Standard Classification of Occupations (ISCO-88) and classified as high-skilled white-collar occupation, low-skilled white- collar occupation, high-skilled blue- collar occupation and low-skilled blue- collar occupation [26]. For patients where a response was difficult to classify, we set a fifth category of unknown.

**Table 1:** Baseline characteristics for patients on graded (20–95%) and full (100%) sickness absence

	Total, *N* = 3371	GSA total, *n* = 1671 (49.6%)	FSA, *n* = 1700 (50.4%)	*P* for diff
*Sociodemographic characteristics*				
**Sex**				< 0.05
Male	1469 (43.6)	596 (35.7)	873 (51.4)	
Female	1902 (56.4)	1075 (64.3)	827 (48.6)	
**Age**				< 0.05
18–29 years	451 (13.4)	196 (11.7)	255 (15.0)	
30–39 years	959 (28.5)	447 (26.8)	512 (30.1)	
40–49 years	1038 (30.8)	555 (33.2)	483 (28.4)	
50–62 years	923 (27.4)	473 (28.3)	450 (26.5)	
Mean (SD)	42.1 (10.4)	42.6 (10.1)	41.7 (10.6)	< 0.05
**Marital status**				< 0.05
Married/ De facto	2533 (75.1)	1294 (77.4)	1239 (72.9)	
Single	838 (24.9)	377 (22.6)	461 (27.1))	
**Educational level**				< 0.05
Primary school	359 (10.6)	146 (8.7)	213 (12.5)	
Vocational school	1324 (39.3)	599 (35.8)	725 (42.7)	
Senior high school	424 (12.6)	200 (12.0)	224 (13.2)	
University 4 years or less	669 (19.8)	365 (21.8)	304 (17.9)	
University over 4 years	595 (17.7)	361 (21.7)	234 (13.7)	
**Occupation (ISCO)**				< 0.05
High skilled white collar	1209 (35.9)	707 (42.3)	502 (29.5)	
Low skilled white collar	983 (29.1)	518 (31.0)	465 (27.3)	
High skilled blue collar	429 (12.7)	158 (9.5)	271 (16.0)	
Low skilled blue collar	370 (11.0)	112 (6.7)	258 (15.2)	
Unknown category	380 (11.3)	176 (10.5)	204 (12.0)	
*Lifestyle characteristics*				
**Smoking daily**				< 0.05
No	2804 (83.2)	1422 (85.1)	1382 (81.3)	
Yes	567 (16.8)	249 (14.9)	318 (18.7)	
**Physical activity**				< 0.05
Inactive	362 (10.7)	152 (9.1)	210 (12.3)	
Active	3009 (89.3)	1519 (90.9)	1490 (87.7)	
*Health measures*				
**Duration of pain**				0.66
Up to 1 year	1780 (52.8)	876 (52.4)	904 (53.2)	
1 year or more	1591 (47.2)	795 (47.6)	796 (46.8)	
**Diagnosis at clinic**				< 0.05
Neck related condition	1659 (49.2)	874 (52.3)	785 (46.2)	
Back related condition	1712 (50.8)	797 (47.7)	915 (53.8)	
**Pain rating rest**	5.4 (2.2)	5.1 (2.2)	5.6 (2.2)	< 0.05
**Pain rating activity**	6.6 (2.0)	6.2 (2.0)	6.9 (1.9)	< 0.05
**ODI/ NDI** Mean (SD)	37.2 (14.1)	34.0 (12.6)	40.3 (14.7)	< 0.05
**FABQ -physical activity** Mean (SD)	12.9 (5.8)	11.7 (5.5)	14.0 (5.8)	< 0.05
**FABQ -work** Mean (SD)	24.7 (9.9)	21.6 (9.3)	27.7 (9.6)	< 0.05
**HSCL10** Mean (SD)	2.0 (0.6)	2.0 (0.6)	2.1 (0.6)	< 0.05
**Number of pain regions** Mean (SD)	6.6 (5.4)	6.6 (5.2)	6.7 (5.5)	0.46
*Duration of sickness absence*				
**Sickness absence before t0**				0.39
1–91 days	1535 (45.5)	769 (46.0)	766 (45.1)	
92–153 days	887 (26.3)	443 (26.5)	444 (26.1)	
154–213 days	464 (13.8)	236 (14.1)	228 (13.4)	
> 213 days	485 (14.4)	223 (13.4)	262 (15.4)	
Mean (SD)	117.3 (83.8)	116.4 (81.2)	118.2 (86.2)	0.54

Categorical variables presented as n (%) and continuous variables presented as mean (SD). SD: Standard deviation. Test statistics: Independent t-test, Welch`s t-test (continuous variables), Chi-squared tests of independence (categorical variables). ISCO: International Classification of Occupations. ODI: Oswestry Disability Index. NDI: Neck Disability Index. FABQ: Fear Avoidance Beliefs Questionnaire. HSCL10: Hopkins Symptom Checklist 10

#### Lifestyle variables

Physical activity was measured by the Saltin-Grimby Physical Activity Level Scale, a 4-level scale where lower levels indicate lower physical activity and consolidated into a binary variable; inactive or active. Daily smoking was reported as a binary variable.

### Duration of SA at baseline

The continuous duration of the current SA spell at the time of assessment was retrieved from NAV register. Participants were categorised into 4 groups: 1–91 days, 92–153 days, 154–213 days and > 213 days [22]. SA could have varied in grading within the period.

### Health measures

The scales used for measuring the patient’s health status were selected by the NNRR, for the purpose of quality assessment of secondary care treatment for patients with MSD. All validated scales were used as continuous scores, rather than cut-of values, to minimize residual confounding.

Intensity of pain during activity and rest were measured by a numeric rating scale, reported in the range from 0 (no pain) to 10 (worst possible pain).

Disability was measured using either the Oswestry Disability Index (ODI) for low back pain and/or the Neck Disability Index (NDI) for neck pain. Each scale contains 10 items rated from 0 to 5. Total scores are converted to a percentage, where 0% indicating no disability and 100% indicating the highest disability level. In cases where both questionnaires were completed by the patient, the higher score was used in analysis [27].

Symptoms of anxiety and/or depression was measured by the Hopkins Symptom Checklist 10 (HSCL10), consisting of 10 items scored from 1 (not at all) to 4 (very much). The responses are summed and divided by 10 (total range: 1.0–4.0), with higher scores indicating greater symptom severity.

Fear-avoidance beliefs were measured by The Fear-Avoidance Belief Questionnaire (FABQ). Each item is rated in the range from 0 (strongly disagree) to 6 (strongly agree). The FABQ-Work subscale consists of 7 items with a score range of 0–42, while the FABQ-Physical Activity subscale has 4 items with a score range of 0–24. Higher scores indicate stronger fear-avoidance beliefs.

Further, pain duration as a binary categorical variable and number of painful areas as a continuous variable were included in the analyses. Clinician data were used for diagnosis, grouped into either neck or back- related.

To preserve participants’ responses and retain sample size/power, we permitted up to 25% missing data in psychometric measurements using missing imputation by the individual patient’s average on valid items. Responses on a specific scale with less than 75% completion were excluded from the analysis [28].

### Statistical analysis

Descriptive statistics were used for baseline characteristics. Group size, normality and equality of variance were assessed. Baseline measurements of the included variables were compared according to SA status at time of assessment, using Chi-square tests for categorical variables and one-way ANOVA or t-tests for continuous variables. Independent t-tests were used when assumptions were met, while Welch`s t-test was applied for variables with unequal variances.

Based on SA status, modified Poisson regression was used to estimate the relative risk (RR) of RTW 12 months after baseline, using robust standard errors to account for variance estimation. Four different predictors were included in two regression analyses; in the first analysis GSA total was compared to FSA, and in the second analysis we compared GSA with low SA-, GSA with middle SA-, and GSA with high SA, to FSA as the reference. Crude and adjusted RR estimates with 95% CI were calculated. For all the predictors we included the adjustment variables in five regression models: (Model 1) unadjusted, (Model 2) model 1 and sociodemographic variables, (Model 3) model 2 and lifestyle variables, (Model 4) model 3 and duration of SA at baseline and (Model 5) model 4 and health measures. Correlations between independent variables were assessed with Pearson’s r for continuous variables, point-biserial correlation for binary categorical variables, and Spearman`s rho for ordinal or non-linear relationships, and none of the correlations exceeded 0.5. All assumptions were assessed and considered met. The RR was reported with 95% confidence intervals for all analyses, and statistical significance was interpreted based on whether the 95% CI crossed 1.0, consistent with a significance level of *p* < 0.05. Data management and linkage of data sources were performed using IBM SPSS Statistics for Windows, Version 29.0 (IBM Corp. Armonk, NY) and statistical analysis were conducted using Stata MP 17 (StataCorp. 2023. Stata Statistical Software: Release 17. College Station, TX: StataCorp LLC).

## Results

Table 1 presents baseline characteristics for the sample of patients (*n* = 3371); GSA total (*n* = 1671, 49.6%) and FSA (*n* = 1700, 50.4%). There were more women (56.4%) than men in the sample, and more women (64.3%) were on GSA. The mean age was highest in the GSA group (42.6 (10.1)), compared to the FSA group (41.7 (10.6)). Education levels were slightly different, where patients on GSA had a higher level of education than the FSA group. Most of the patients (45.5%) had been on SA between 1 and 91 days at the time of assessment. Patients on FSA reported more severe symptoms than patients on GSA according to all included measures.

Table 2 presents baseline characteristics according to detailed levels of GSA; GSA with low SA ( < = 40%, *n* = 391 (23.4%)), GSA with middle SA (> 40% and < = 60%, *n* = 902 (54.0%)), and GSA with high SA (> 60% and < 100%, *n* = 378 (22.6%)), with the mean values for the symptom scales indicating more severe health symptoms with higher levels of SA grading.

**Table 2:** Baseline characteristics according to levels of graded sickness absence

	GSA with low SA ( < = 40%), *n* = 391 (23.4%)	GSA with middle SA (> 40% and < = 60%), *n* = 902 (54.0%)	GSA with high SA (> 60% and < 100%), *n* = 378 (22.6%)	*P* for diff
*Sociodemographic characteristics*				
**Sex**				< 0.05
Male	102 (26.1)	361 (40.0)	133 (35.2)	
Female	289 (73.9)	541 (60.0)	245 (64.8)	
**Age**				0.34
18–29 years	45 (11.5)	107 (11.9)	44 (11.6)	
30–39 years	104 (26.6)	231 (25.6)	112 (29.6)	
40–49 years	144 (36.8)	290 (32.2)	121 (32.0)	
50–62 years	98 (25.1)	274 (30.4)	101 (26.7)	
Mean (SD)	42.4 (9.7)	43.0 (10.4)	42.0 (9.7)	0.23
**Marital status**				0.22
Married/ De facto	312 (79.8)	684 (75.8)	298 (78.8)	
Single	79 (20.2)	218 (24.2)	80 (21.2)	
**Educational level**				< 0.05
Primary school	29 (7.4)	83 (9.2)	34 (9.0)	
Vocational school	107 (27.3)	346 (38.3)	146 (38.6)	
Senior high school	46 (11.8)	107 (11.9)	47 (12.4)	
University 4 years or less	91 (23.3)	193 (21.4)	81 (21.5)	
University over 4 years	118 (30.2)	173 (19.2)	70 (18.5)	
**Occupation (ISCO)**				< 0.05
High skilled white collar	205 (52.4)	357 (39.6)	145 (38.3)	
Low skilled white collar	106 (27.1)	276 (30.1)	136 (36.0)	
High skilled blue collar	27 (6.9)	102 (11.3)	29 (7.7)	
Low skilled blue collar	9 (2.3)	76 (8.4)	27 (7.1)	
Unknown category	44 (11.3)	91 (10.1)	41 (10.9)	
*Lifestyle characteristics*				
**Smoking daily**				0.49
No	338 (86.5)	759 (84.2)	325 (86.0)	
Yes	53 (13.5)	143 (15.8)	53 (14.0)	
**Physical activity**				0.20
Inactive	35 (9.0)	74 (8.2)	43 (11.4)	
Active	356 (91.0)	828 (91.8)	335 (88.6)	
*Health measures*				
**Duration of pain**				0.32
Up to 1 year	200 (51.1)	465 (51.6)	211 (55.8)	
1 year or more	191 (48.9)	437 (48.4)	167 (44.2)	
**Diagnosis at clinic**				0.75
Neck related condition	208 (53.2)	464 (51.4)	202 (53.4)	
Back related condition	183 (46.8)	438 (48.6)	176 (46.6)	
**Pain rating rest**	5.0 (2.2)	5.2 (2.2)	5.2 (2.1)	0.29
**Pain rating activity**	5.9 (2.0)	6.2 (2.0)	6.5 (2.0)	< 0.05
**ODI/ NDI** Mean (SD)	32.2 (11.8)	33.8 (12.7)	36.5 (12.8)	< 0.05
**FABQ -physical activity** Mean (SD)	10.8 (5.5)	11.8 (5.4)	12.4 (5.7)	< 0.05
**FABQ -work** Mean (SD)	18.1 (9.3)	21.8 (9.0)	24.8 (9.0)	< 0.05
**HSCL10** Mean (SD)	1.9 (0.6)	2.0 (0.6)	2.0 (0.6)	0.09
**Number of pain regions** Mean (SD)	6.2 (5.0)	6.8 (5.3)	6.4 (5.1)	0.21
*Duration of Sick leave*				
**Sickness absence before t0**				< 0.05
1–91 days	184 (47.1)	446 (49.5)	139 (36.8)	
92–153 days	108 (27.6)	230 (25.5)	105 (27.8)	
154–213 days	47 (12.0)	119 (13.2)	70 (18.5)	
> 213 days	52 (13.3)	107 (11.8)	64 (16.9)	
Mean (SD)	114.6 (82.7)	110.0 (80.6)	133.6 (79.0)	< 0.05

Categorical variables presented as n (%) and continuous variables presented as mean (SD). SD: Standard deviation. Test statistics: one-way ANOVA (continuous variables), Chi-squared test of independence (categorical variables). ISCO: International Classification of Occupations. ODI: Oswestry Disability Index. NDI: Neck Disability Index. FABQ: Fear Avoidance Beliefs Questionnaire. HSCL10: Hopkins Symptom Checklist 10

At 12-month follow-up 1107 (66.2%) patients in the GSA total group and 871 (51.2%) patients in the FSA group had returned to work. Within the different levels of GSA, 307 (78.5%) of the patients in GSA with low SA, 585 (64.9%) in GSA with middle SA and 215 (56.9%) in GSA with high SA had returned to work.

In Table 3 we present the regression models for GSA total versus FSA and RTW. The unadjusted association (RR) between GSA total and RTW was 1.29 (95% CI 1.22–1.37). Both adjusting for sociodemographic factors (RR = 1.30, 95% CI 1.22–1.38), and further adjustments for lifestyle variables (RR = 1.29, 95% CI 1.22–1.37) and duration of SA at baseline (RR = 1.29, 95% CI 1.22–1.36) did little to the association. In the fully model including all health measures, the benefit of GSA total over FSA was somewhat attenuated but remained statistically significant (RR = 1.19, 95% CI 1.12–1.26). All models for GSA total were significant at a level of *p* < 0.05.

**Table 3:** Poisson regression models for GSA total (20–95% SA) and RTW at 12-month follow-up, with full sickness absence as reference, *n* = 3371

Regression models	RR (95% CI)	*P* value
Model 1: unadjusted	1.29 (1.22–1.37)	< 0.05
Model 2: model 1 + sociodemographic variables^a^	1.30 (1.22–1.38)	< 0.05
Model 3: model 2 + lifestyle variables^b^	1.29 (1.22–1.37)	< 0.05
Model 4: model 3 + duration of sickness absence at baseline^c^	1.29 (1.22–1.36)	< 0.05
Model 5: model 4 + health measures^d^	1.19 (1.12–1.26)	< 0.05

^a^ Sociodemographic variable: sex, age, marital status, educational level, occupation

^b^ Lifestyle variables: smoking, physical activity

^c^ Categorized into 4 groups: 1–91 days, 92–153 days, 154–213 days, > 213 days

^d^ Health measures: duration of pain, pain rating rest, pain rating activity, ODI/NDI score, diagnosis at clinic, FABQ Physical Activity, FABQ Work, HSCL10, number of pain regions

The RRs and 95% CI for all adjustment variables included in the full model for the association between GSA total versus FSA and RTW can be inspected in Supplementary Table 1 (S1).

Analysis with GSA as a predictor in a more detailed manner, revealed that the effect varied across levels of GSA. In all models, the association was strongest for GSA with low SA, slightly lower at GSA with middle SA, and lowest for GSA with high SA. In the fully adjusted model, the GSA with high SA was no longer significantly associated with RTW (*p* = 0.07).

**Table 4:** Poisson regression models for levels of GSA and RTW at 12-month follow-up, with full sickness absence as reference, *n* = 3371

	GSA with low SA ( < = 40%), *n* = 391		GSA with middle SA (> 40% and < = 60%), *n* = 902		GSA with high SA (> 60% and < 100%), *n* = 378	
Regression models	RR (95% CI)	*P* value	RR (95% CI)	*P* value	RR (95% CI)	*P* value
Model 1: GSA, unadjusted	1.53 (1.43–1.64)	< 0.05	1.27 (1.43–1.64)	< 0.05	1.11 (1.01–1.23)	< 0.05
Model 2: model 1 + sociodemographic variables^a^	1.53 (1.42–1.64)	< 0.05	1.28 (1.19–1.37)	< 0.05	1.12 (1.02–1.24)	< 0.05
Model 3: model 2 + lifestyle variables^b^	1.53 (1.42–1.64)	< 0.05	1.27 (1.19–1.36)	< 0.05	1.12 (1.01–1.23)	< 0.05
Model 4: model 3 + duration of sick leave at baseline^c^	1.52 (1.41–1.63)	< 0.05	1.25 (1.17–1.34)	< 0.05	1.15 (1.04–1.26)	< 0.05
Model 5: model 4 + health measures^d^	1.35 (1.25–1.46)	< 0.05	1.17 (1.09–1.25)	< 0.05	1.09 (0.99–1.20)	0.07

^a^ Sociodemographic variable: sex, age, marital status, educational level, occupation

^b^ Lifestyle variables: smoking, physical activity

^c^ Categorized into 4 groups: 1–91 days, 92–153 days, 154–213 days, > 213 days

^d^ Health measures: duration of pain, pain rating rest, pain rating activity, ODI/NDI score, diagnosis at clinic, FABQ Physical Activity, FABQ Work, HSCL10, number of pain regions

## Discussion

In this longitudinal cohort study among patients referred to a physical medicine outpatient clinic, the patients on FSA had consistently higher symptom levels than patients on GSA according to all included measures. Furthermore, the GSA group had higher socioeconomic status than the FSA group, both in terms of occupations and educational level. Despite these differences, GSA total was more strongly associated with RTW than FSA both with and without adjustment for potential confounding from health and socioeconomic differences between the groups. Furthermore, the association with RTW varied across levels of GSA, with consistently higher RRs observed among those in the group GSA with low SA. To the best of our knowledge, this is the first time an analysis of the relationship between GSA and RTW has included the potentially confounding effects of self-reported health.

Overall, SA status at baseline corresponds with the patient’s self- reported severity of symptoms, with higher levels of GSA associated with more severe symptoms. This supports the intention for the GSA policy; patients prescribed GSA are unable to work full time, but remain capable of working in a reduced capacity, and that the levels of GSA follow symptom severity [14]. In the final adjusted model, low and middle levels of GSA remained significantly associated with RTW, suggesting that neither sociodemographic variables, lifestyle variables or the severity of health problems fully explain the relationship between GSA and RTW. This observation aligns with Bosman`s study, which found that none of the included confounders significantly attenuated the association between GSA and SA duration [17]. However, our findings indicate the weakest association between GSA with high SA and RTW, compared to FSA. This group is characterised by both having the most severe symptoms and the lowest participation in work. For patients with MSD of such severity that they are referred to specialist healthcare services, the goal is to restore functional ability. However, RTW as a goal might be most relevant for those with the least severe symptoms, such as mild to moderate musculoskeletal disorders [29]. It has been suggested that mild to moderate conditions are defined more by individuals ‘responses to the illness and symptoms, highlighting a considerable opportunity for RTW outcomes [30] compared to those with more severe symptoms. However, from a perspective where SA is regarded as a treatment [16], the findings may suggest a dose-response pattern, implying that a certain amount of work participation is necessary to facilitate RTW. Among patients on GSA with high SA, where working hours are low, the “dose” may be insufficient to facilitate the hypothesised beneficial effects associated with work participation on RTW.

Work characteristics beyond occupation category may influence the effectiveness of GSA in facilitating RTW, and the opportunity for GSA might not be the same for all patients. For instance, patients with more severe or complex conditions may be less likely to receive GSA if the possibility for workplace accommodations are limited, while those who present with milder symptoms or more flexible job conditions may be more likely to receive GSA. Low-skilled blue-collar occupations are a recognized risk factor for long-term SA because of low back pain [31].

Further adding to the complexity are the numerous factors associated with RTW (S1). The duration of SA at baseline was significantly associated with RTW, which has been confirmed previously in patients with chronic musculoskeletal pain [22]. In our results, the strongest association between GSA and RTW relative to FSA was observed for the longest duration of SA. From a point of view where SA is used as a treatment the RTW process is gradual and stepwise, as an essential intervention to rebuild work capacity, especially for those with prolonged SA durations. Interpreting the impact of SA duration on RTW is nevertheless difficult, as it could represent both the severity of the illness, adaption to reduced function, time to recovery and/or the influence of the compensation system which in turn depends on the national welfare systems. Further, in line with previous studies, we found that lower levels of education were associated with a higher proportion of SA [32], and that education levels were significantly associated with RTW. Additionally, consistent with studies examining predictors for SA due to pain, our findings showed that age, duration of pain, number of pain regions [33], fear avoidance beliefs [31, 33], neck or back disability scores (ODI/NDI) [31] and symptoms of anxiety and/or depression (HSCL10) [22] were statistically significant associated with RTW outcomes in the patient population.

The overall benefits of GSA on RTW is in line with several studies, which have concluded with a beneficial effect of graded exposure to work on work participation, either in terms of reduced risk of permanent work disability [23, 24], of increased work participation [34–36], faster and more sustainable RTW [37], increased probability of RTW [8, 38] or shorter work absences [39], though comparing the results should be done with caution, as multiple factors differ. Notably, the diagnosis, methods and setting differ across the studies. The causal mechanisms underlying this association remains unclear, but multiple hypotheses have been proposed. First, some work-related activity (as achieved with GSA) may be beneficial for health, thereby increasing the probability of RTW compared to no work as in FSA (7–8, 15). Second, it is also plausible that maintaining a connection to work could positively impact an individual’s illness perceptions and behaviour, potentially playing a role in facilitating RTW [39, 40]. Finally, several studies emphasise the individual self-efficacy for RTW (i.e. confidence in task success) as a strong prognostic factor, suggesting patients on GSA exhibit higher self-efficacy than those on FSA [41, 42].

Our findings expand the understanding of how GSA is associated with RTW, and how this association is attenuated according to several factors. However, further research is needed to explore any potential causal mechanisms for the benefits of GSA over FSA on RTW. Since the context of the present study is patients suffering from MSD, we can only speculate as to whether the observed associations between GSA and RTW remain for other patient groups. The findings provide empirical support to policies in many countries advocating GSA over FSA to promote RTW. Research on how to promote GSA over FSA, and to identify eligible patients for the measure is also needed. While several countries have a policy for GSA, the uptake of GSA in the Nordic countries has been more successful, for example, than the Fit Note system in the UK. Potential long-term side-effects of GSA should also be investigated.

### Strengths and limitations

An important strength of the study is the use of multiple self-reported health data applied as scale-scores to prevent residual confounding. The self-reported data was collected from patients in a naturalistic setting without any incentives, with a relatively high participation rate. The use of registry data for exposure and outcome improves reliability and prevents attrition. The study was also adequately powered for this purpose. However, the cohort comprises patients with MSD eligible for assessment in specialist outpatient secondary health care, indicative of severity and chronicity of health problems. This limits generalizability to healthier patient groups. Another limitation was the number of missing items in some of the questionnaires. Even with our comprehensive adjustments for symptom severity, the issue of residual confounding cannot be dismissed. Though our study adjusts for a broad range of clinically relevant self-reported health measures to assess the severity of the complaints, there may be unmeasured aspects of health that are relevant for the association. For example, even though the analysis is adjusted for symptoms of anxiety and /or depression as measured by HSCL10, patients with chronic musculoskeletal pain frequently presents with such co-occurrent symptoms and this is associated with greater functional limitations than either condition alone [43]. Examples of other unmeasured aspects may include work-related factors beyond occupation category, possibly contributing to the association between GSA and RTW, as well as other unmeasured characteristics of the different levels of GSA. While this study aimed to explore the impact of health in the association between GSA and RTW, the direction of the observed associations should still be interpreted with caution due to the limitations of the observational study design.

## Conclusions

In this sample of sick listed patients referred to a physical medicine outpatient clinic, patients on GSA differ from patients on FSA with respect to symptom severity and sociodemographic factors. We find benefits of GSA compared to FSA, on RTW both with and without adjustment for these factors. While causal mechanisms for this observed association are largely unknown, our study lends empirical support to policies aiming to promote GSA over FSA.

## Electronic supplementary material

Below is the link to the electronic supplementary material.

Supplementary Material 1

## Data Availability

The data supporting this paper may be shared upon application to the scientific board of the NNRR and NAV and requires an ethical approval from REC North.

## Declarations

### Other

AI was used to improve language.

## Abbreviations

DP: Disability Payments
FABQ: The Fear-Avoidance Belief Questionnaire
FSA: Full time sickness absence
GSA: Graded sickness absence
HSCL10: Hopkins Symptom Checklist 10
ISCO-88: International Standard Classification of Occupations
MSD: Musculoskeletal disorders
NAV: The Norwegian Labour and Welfare Administration
NDI: Neck Disability Index
NNRR: Norwegian Neck and Back Registry
ODI: Oswestry Disability Index
RTW: Return to work
SA: Sickness absence
WAA: Work Assessment Allowance
