# Supplementary material for: Is the association between graded sickness absence and return to work confounded by health? A longitudinal cohort study from the Norwegian neck and back registry
Source: BMC Public Health. 2025 Mar 30;25:1202. doi: 10.1186/s12889-025-22368-1 (PMC11956489; doi:10.1186/s12889-025-22368-1)
Supplement: Supplementary file 1 — Supplementary Material 1 [file 12889_2025_22368_MOESM1_ESM.docx]

*Supplementary Table 1: RTW at 12-month follow-up, GSA total vs FSA, n= 3371, all covariates*

| Regression model 5 | RR (95% CI) | P- value | Overall P- value (for categorical) |
| --- | --- | --- | --- |
| Sickness Absence (SA) |  |  | <0.05 |
| FSA (100% SA) | 1.0 (ref) |  |  |
| GSA total (20-95% SA) | 1.19 (1.12-1.26) | <0.05 |  |
| Sex |  |  | 0.31 |
| Male | 1.0 (ref) |  |  |
| Female | 0.97 (0.91-1.03) | 0.31 |  |
| Age | 0.99 (0.99-1.00) | <0.05 |  |
| Marital status |  |  |  |
| Married/cohabitant | 1.0 (ref) |  | 0.37 |
| Single | 1.03 (0.97-1.09) | 0.37 |  |
| Education level |  |  | <0.05 |
| Primary School | 1.0 (ref) |  |  |
| Vocational School | 1.29 (1.13-1.47) | <0.05 |  |
| Senior High School | 1.35 (1.17-1.55) | <0.05 |  |
| University 4 years or less | 1.36 (1.19-1.56) | <0.05 |  |
| University over 4 years | 1.35 (1.17-1.55) | <0.05 |  |
| Occupation (ISCO) |  |  | 0.10 |
| High skilled white collar | 1.0 (ref) |  |  |
| Low skilled white collar | 0.95 (0.88-1.02) | 0.17 |  |
| High skilled blue collar | 1.06 (0.95-1.17) | 0.29 |  |
| Low skilled blue collar | 1.00 (0.89-1.12) | 0.98 |  |
| Unknown category | 1.06 (0.97-1.16) | 0.17 |  |
| Smoking |  |  | 0.47 |
| No | 1.0 (ref) |  |  |
| Yes | 0.97 (0.89-1.05) | 0.47 |  |
| Physical activity |  |  | 0.64 |
| Inactive | 1.0 (ref) |  |  |
| Active | 1.02 (0.93-1.13) | 0.64 |  |
| Duration of SA at baseline |  |  | <0.05 |
| 1-91 days | 1.0 (ref) |  |  |
| 92-153 days | 0.95 (0.89 -1.01) | 0.09 |  |
| 154-213 days | 0.77 (0.70-0.85) | <0.05 |  |
| >213 days | 0.64 (0.58-0.72) | <0.05 |  |
| Duration of pain |  |  | <0.05 |
| < 1 year | 1.0 (ref) |  |  |
| 1 year or more | 0.90 (0.85-0.95) | <0.05 |  |
| Pain rating rest | 1.00 (0.98-1.01) | 0.84 |  |
| Pain rating activity | 0.99 (0.98-1.01) | 0.42 |  |
| ODI/NDI score | 1.00 (0.99-1.00) | <0.05 |  |
| Diagnosis at clinic |  |  | 0.92 |
| Neck-related | 1.0 (ref) |  |  |
| Back-related | 1.00 (0.95-1.06) | 0.92 |  |
| FABQ Physical Activity | 1.01 (1.00-1.01) | <0.05 |  |
| FABQ Work | 0.99 (0.99-0.99) | <0.05 |  |
| HSCL10 | 0.88 (0.83-0.93) | <0.05 |  |
| Number of pain regions | 0.99 (0.98-1.00) | <0.05 |  |

*SA: Sickness Absence. FSA: Full Sickness Absence. GSA: Graded Sickness Absence. ISCO: International Classification of Occupations. ODI: Oswestry Disability Index. NDI: Neck Disability Index. FABQ: Fear Avoidance Beliefs Questionnaire. HSCL10: Hopkins Symptom Checklist 10.*
